# Epidemic and virulence characteristic of *Shigella* spp. with extended-spectrum cephalosporin resistance in Xiaoshan District, Hangzhou, China

**DOI:** 10.1186/1471-2334-14-260

**Published:** 2014-05-15

**Authors:** Chuan-Ling Zhang, Qing-Zhong Liu, Juan Wang, Xu Chu, Li-Meng Shen, Yuan-Yu Guo

**Affiliations:** 1 Department of Clinical Laboratory, Zhejiang Xiaoshan Hospital, Zhejiang Province, China; 2 Department of Clinical Laboratory, Shanghai First People’s Hospital, School of Medicine, Shanghai Jiaotong University, 100 Haining Road, Shanghai 200080, People’s Republic of China

**Keywords:** *Shigella*, Extended-spectrum cephalosporin, Resistance, Cephalosporin resistance mechanism, Virulence markers, Genotyping

## Abstract

**Background:**

*Shigellae* have become increasingly resistant to the extended-spectrum cephalosporin (ESC) worldwide and pose a great challenge to anti-infection treatment options. The purpose of this study was to determine the resistance, cephalosporin resistance mechanisms, virulence characteristic and genotype of ESC-resistant *Shigella*.

**Methods:**

From 2008 to 2012, *Shigella* isolates collected from diarrhea patients were detected for antibiotics sensitivity by disk diffusion, cephalosporin resistance determinants and virulence genes using polymerase chain reaction (PCR) and genotyping through enterobacterial repetitive intergenic consensus sequence PCR (ERIC-PCR).

**Results:**

A total of 356 *Shigella* isolates were gathered, and 198 (55.6%, 58 *S. flexneri* and 140 *S. sonnei*) were resistant to ESC. All ESC-resistant isolates were susceptible to imipenem, and only 0.5% isolate was resistant to piperacillin/tazobactam. ESC-resistant *S. flexneri* showed high degrees of resistance to ampicillin (100%), ampicillin/sulbactam (96.6%), piperacillin (100%), trimethoprim/sulfamethoxazole (74.1%), ciprofloxacin (74.1%), levofloxacin (53.4%), ceftazidime (58.6%) and cefepime (58.6%). ESC-resistant *S. sonnei* exhibited high resistance rates to ampicillin (100%), piperacillin (100%) and trimethoprim/sulfamethoxazole (96.4%). Cephalosporin resistance genes were confirmed in 184 ESC-resistant isolates. *bla*_*CTX-M*_ types (91.8%, mainly *bla*_*CTX-M-14*_, *bla*_*CTX-M-15*_ and *bla*_*CTX-M-57*_) were most prevalent, followed by *bla*_*OXA-30*_ (26.3%). Over 99.0% ESC-resistant isolates harbored virulence genes *ial*, *ipaH*, *virA* and *sen*. However, *set1* were more prevalent in ESC-resistant *S. flexneri* isolates than in *S. sonnei* isolates. ERIC-PCR results showed that 2 and 3 main genotypes were detected in ESC-resistant *S. flexneri* and *S. sonnei*, respectively.

**Conclusion:**

Our findings indicated that a high prevalence of ESC-resistant *Shigella* mediated mainly by *bla*_*CTX-M*_ with stronger resistance and virulence, and the existence of specific clones responsible for these *Shigella* infection in the region studied.

## Background

Shigellosis continues to be a major public health problem in non-industrialized countries. It was reported that about 91 million cases of shigellosis occurred, with 414,000 people dying in Asia annually [1]. In China, the morbility of *Shigella* infections each year is unusually high. Wang et al. [2] have reported that in 2000 0.8-1.7 million episodes of shigellosis occurred in seven different geographical regions, China.

For shigellosis, antibiotic therapy can reduce the duration and severity of the illness [3]. However, with the long-term overuse of antimicrobials, the resistant *Shigella* has prevailed all over the word, and production of extended-spectrum β-lactamases (ESBLs) or AmpC enzymes is one of the most important resistance mechanisms. In China extended-spectrum cephalosporin (ESC)-resistant *Shigella* spp. has also been described in recent years [3-7]. However, as China has a vast territory area, the spectrum of antimicrobial resistance and the mechanisms of resistance may vary in different regions. Therefore, it is necessary to explore the variations of ESC resistance in clinical isolates of *Shigella* spp. from different geographical areas.

The ability of *Shigella* spp. to cause shigellosis is attributed to the expression of arrays of virulence genes associated with colonization, invasion/penetration and toxin-mediated disease, such as the invasion-associated locus (*ial*), the invasion plasmid antigen H gene (*ipaH*), *Shigella* enterotoxin 1 (ShET-1) gene (*set1A* and *set1B*), *Shigella* enterotoxin 2 (ShET-2) gene (*sen*) and *virA* gene [8,9]. Estimating the existence of virulence determinants in *Shigella* would help us better understand its pathogenicity. However, investigations into clinical *Shigella* spp. virulence factors are still rare in the world.

The purpose of this study was to investigate the resistance, the cephalosporin resistance mechanisms and the prevalence of putative virulence genes in ESC-resistant *Shigella* from patients with dysentery in Xiaoshan District, suburban of Hangzhou city, Zhejiang province, China. Finally we further ascertained the genotypes of these strains by ERIC-PCR (enterobacterial repetitive intergenic consensus sequence PCR) typing.

## Methods

### Bacterial isolates

Between January 2008 and December 2012, a total of 356 nonduplicate *Shigella* isolates were obtained from the faeces of different diarrhea patients at Zhejiang Xiaoshan hospital, Hangzhou City, Zhejiang Province, China. All isolates were identified by VITEK 60 microbial identification system (BioMérieux Corp., Lyon, France) and serotyped using commercial antisera (Lanzhou Institute of Biological Products, Lanzhou, China). Isolates studied were taken as part of standard patient care. Because of being focused on bacteria, no ethical approval was required for this study.

### Antimicrobial susceptibility testing

The *Shigella* isolates were detected for antibiotic susceptibility by Kirby–Bauer method for the following antibiotics (Oxoid, Hampshire, UK), according to Clinical and Laboratory Standards Institute (CLSI), 2012 [10]: ampicillin (AMP), piperacillin (PIP), ciprofloxacin (CIP), trimethoprim/sulfamethoxazole (SXT), levofloxacin (LEV), ampicillin/sulbactam (SAM), piperacillin/tazobactam (TZP), imipenem (IMP), cefotaxime (CTX), ceftazidime (CAZ), cefepime (FEP) and cefoxitin (FOX). ESC-resistant organisms were defined as isolates showing resistance or intermediate to cefotaxime (zone diameter ≤25 mm) or ceftazidime (zone diameter ≤20 mm) or cefepime (zone diameter ≤17 mm) [10]. *Escherichia coli* ATCC 25922 was used for antibiotic susceptibility testing quality control.

### ESBL and AmpC detection

ESC-resistant isolates were screened for ESBL production by the phenotypic confirmatory test according to CLSI [10]. AmpC examination was performed by the three-dimensional extract test as described by Philip et al. [11] for cefoxitin resistant or intermediate isolates.

### ESBL, AmpC and virulence genes amplification

Detection of the ESBL genes (*bla*_*TEM*_, *bla*_*OXA*_, *bla*_*CTX-M-1*, *2*, *8*, *9*_ and *bla*_*SHV*_) and virulence determinants (*ial*, *iapH*, *set1A*, *set1B*, *sen* and *virA*) were performed by PCR for ESC-resistant isolates, as described previously [3,12,13]. AmpC genes (*bla*_*MOX*_, *bla*_*CIT*_, *bla*_*DHA*_, *bla*_*ACC*_, *bla*_*EBC*_ and *bla*_*FOX*_) were examined by multiplex-PCR for cefoxitin resistant or intermediate isolates according to Perez-Perez et al. [14]. Sequences of the primers were given in Table 1. The PCR products for ESBL and AmpC genes were sequenced on both DNA strands with ABI 3730XL sequencer (Perkin-Elmer) by Shanghai Sangon Biotech. The nucleotide sequences were analyzed by the BLAST program. For virulence genes, a representative amplicon for the each gene detected was also sequenced to validate that primers amplified the target genes.

**Table 1:** **Primers used for PCR to detect *Shigella *** **resistance and virulence genes**

**Primer**	**Sequence (5′-3′)**	**Amplicon size (bp)**	**Reference**
ESBL genes			
*CTX-M-1* group	F-GGCCCATGGTTAAAAAATCACTGC	891	[3]
	R-CCGTTTCCGCTATTACAAACCGTTG		
*CTX-M-2* group	F-CTCAGAGCATTCGCCGCTCA	843	
	R-CCGCCGCAGCCAGAATATCC		
*CTX-M-8* group	F-ATGATGAGACATCGCGTTAAC	864	
	R-CGGTGACGATTTTCGCGGCAG		
*CTX-M-9* group	F-GTGACAAAGAGAGTGCAACGG	856	
	R-ATGATTCTCGCCGCTGAAGCC		
*OXA* group	F-ACACAATACATATCAACTTCGC	813	
	R-AGTGTGTTTAGAATGGTGATC		
*SHV* group	F-GCCTTTTCGGCCTTCACTCAAG	989	
	R-TTAGCGTTGCCAGTGCTCGATCA		
*TEM* group	F-ATAAAATTCTTGAAGACGAAA	1076	
	R-GACAGTTACCAATGCTTAATC		
AmpC β-lactamase genes			
*MOX* group	F-GCTGCTCAAGGAGCACAGGAT	520	[14]
	R-CACATTGACATAGGTGTGGTGC		
*CIT* group	F-TGGCCAGAACTGACAGGCAAA	462	
	R-TTTCTCCTGAACGTGGCTGGC		
*DHA* group	F-AACTTTCACAGGTGTGCTGGGT	405	
	R-CCGTACGCATACTGGCTTTGC		
*ACC* group	F- AACAGCCTCAGCAGCCGGTTA	346	
	R- TTCGCCGCA ATCATCCCTAGC		
*EBC* group	F- TCGGTAAAGCCGATGTTGCGG	302	
	R- CTTCCACTGCGGCTGCCAGTT		
*FOX* group	F- AACATGGGGTATCAGGGAGATG	190	
	R- CAA AGCGCGTAACCGGATTGG		
Virulence gene			
*ial*	F-CTGGATGGTATGGTGAGG	320	[12]
	R-GGAGGCCAACAATTATTTCC		
*ipaH*	F-TGGAAAAACTCAGTGCCTCT	423	
	R-CCAGTCCGTAAATTCATTCT		
*setlA*	F-TCACGCTACCATCAAAGA	309	
	R-TATCCCCCTTTGGTGGTA		
*setlB*	F-GTGAACCTGCTGCCGATATC	147	
	R-ATTTGTGGATAAAAATGACG		
*sen*	F-ATGTGCCTGCTATTATTTAT	799	
	R-CATAATAATAAGCGGTCAGC		
*virA*	F-CTGCATTCTGGCAATCTCTTCACATC	215	[13]
	R-TGATGAGCTAACTTCGTAAGCCCTCC		

### ERIC-PCR

ERIC-PCR was carried out on ESC-resistant *Shigella* isolates using primer ERIC2: 5′-AAGTAAGTGACTGGGGTGAGCG-3′, as described by Navia et al. [15].

### Clinical data

Clinical information of the patients was collected from the case notes. The data included date of onset, symptoms (such as times of diarrhea, nausea, bellyache, vomiting, fever and bloody stools), results of microscopic examination of stool (white blood cell and red blood cell), treatment and duration of illness.

### Statistical analysis

Pearson’s chi-square test or Fisher’s exact test if necessary was used to analyze the differences of antibiotics resistance, and virulence genes, and the relationship of virulence factors, resistance and ERIC-PCR genotypes (SPSS 11.5). All statistical tests were two tailed, with *p* < 0.05 considered statistically significant.

## Results

### Distribution of *Shigella* spp

Among 356 *Shigella* isolates, *S. sonnei* was the most prevalent species (n = 244, 68.5%, 244/356), followed by *S. flexneri* (n = 112, 31.5%, 112/356). No sample was positive for *S. dysenteriae* and *S. boydii*. In *S. flexneri*, seven different serotypes were found: 63 type 2a, 15 type 2c, 18 type 4c, 6 type 2b, 5 type 1a, 3 type x and 2 type 3a. 35.7% (40/112) of *S. flexneri* and 47.1% (115/244) of *S. sonnei* cases were found among patients under 5 years old; 20.5% (23/112) of *S. flexneri* and 23.4% (57/244) of *S. sonnei* isolates were from victims aged from 5 to 14 years; 43.8% (49/112) of *S. flexneri* and 29.5% (72/244) of *S. sonnei* isolates were from patients aged ≥15 years.

### Antimicrobial resistance

Of the 356 *Shigella* isolates, 198 (55.6%, 58 *S. flexneri* and 140 *S. sonnei*) were resistant to cefotaxime, 59 (16.6%) to ceftazidime, 66 (18.5%) to cefepime (Table 2). The strains with resistance to ceftazidime or cefepime were all resistant to cefotaxime. Thus, these cefotaxime resistant strains were referred to as ESC-resistant isolates in this study. In addition, in these ESC-resistant isolates, 8 (2.2%) were resistant or intermediate to cefoxitin.

**Table 2:** **Antibiotic resistance of ESC-resistant and susceptible *Shigella *** **isolates**

**Antibiotic**	**Number of isolates resistant to antibiotics (%)**							**ESC-resistant *S. flexneri *** **vs ESC-resistant *S. sonnei*** **(*p *** **value)**
	**Total (n = 356)**	***S. flexneri *** **( n = 112)**			***S. sonnei *** **(n = 244)**			
		**ESC-resistant (n = 58)**	**Cefotaxime susceptible (n = 54)**	**ESC-resistant vs cefotaxime susceptible (*p *** **value)**	**ESC-resistant (n = 140)**	**Cefotaxime susceptible (n = 104)**	**ESC-resistant vs cefotaxime susceptible (*p *** **value)**	
AMP	348 (97.8)	58 (100)	51(94.4)	0.069	140(100)	99(95.2)	0.009	NA
PIP	298 (83.7)	58(100)	46(85.2)	0.002	140(100)	54 (51.9)	<0.001	NA
SAM	136 (38.2)	56(96.6)	46(85.2)	0.035	34(24.3)	0 (0.0)	<0.001	<0.001
TZP	1 (0.3)	1 (1.7)	0 (0.0)	3.332	0 (0.0)	0 (0.0)	NA	0.119
CAZ	59 (16.6)	34(58.6)	0 (0.0)	<0.001	25(17.9)	0 (0.0)	<0.001	<0.001
FEP	66 (18.5)	34(58.6)	0 (0.0)	<0.001	32(22.9)	0 (0.0)	<0.001	<0.001
FOX	8 (2.2)	3 (5.2)	0 (0.0)	0.090	5 (3.6)	0 (0.0)	0.056	0.618
IPM	0(0.0)	0(0)	0 (0.0)	NA	0 (0.0)	0 (0.0)	NA	NA
SXT	304 (85.4)	43(74.1)	25(46.3)	0.003	135(96.4)	101(97.1)	0.766	<0.001
CIP	86 (24.2)	43 (74.1)	39 (72.2)	0.819	3 (2.1)	1 (1.0)	0.485	<0.001
LEV	57 (16.0)	31(53.4)	20(37.0)	0.081	3 (2.1)	3 (2.9)	0.692	<0.001
β-lactam antibiotic + SXT + CIP or LEV	69 (19.4)	36 (62.1)	23 (42.6)	0.039	6 (4.3)	4 (3.8)	0.864	<0.001

AMP, ampicillin; PIP, piperacillin; SAM, ampicillin/sulbactam; TZP, piperacillin/tazobactam; FEP, cefepime; FOX, cefoxitin; IMP, imipenem; SXT, trimethoprim/sulfamethoxazole; CIP, ciprofloxacin; LEV, levofloxacin.

*p* < 0.05 were considered statistically significant.

NA, not available.

Compared to cefotaxime susceptible *S. flexneri* isolates, the ESC-resistant *S. flexneri* isolates were more resistant to PIP (100% versus 85.2%, *p* = 0.002), SAM (96.6% versus 85.2%, *p* = 0.035), SXT (74.1% versus 46.3%, *p* = 0.003), CAZ (58.6% versus 0%, *p* < 0.001) and FEP (58.6% versus 0%, *p* < 0.001). In *S. sonnei*, the ESC-resistant strains were more resistant to AMP (100% versus 95.2%, *p* = 0.009), PIP (100% versus 51.9%, *p* < 0.001), SAM (24.3% versus 0%, *p* < 0.001), CAZ (17.9% versus 0%, *p* < 0.001) and FEP (22.9% versus 0%, *p* < 0.001) than the cefotaxime susceptible isolates (Table 2). Compared to ESC-resistant *S. sonnei* isaolates, the ESC-resistant *S. flexneri* isolates were significantly more likely to be resistant to CAZ (58.6% versus 17.9%, *p* < 0.001), FEP (58.6% versus 22.9%, *p* < 0.001), CIP (74.1% versus 2.1%, *p* < 0.001) and LEV (53.4% versus 2.1%, *p* < 0.001), and less likely to be resistant to SXT (74.1% versus 96.4%, *p* < 0.001). No *Shigella* isolates were resistant to IMP, and Only one *S. flexneri* isolates was resistant to TZP (Table 2).

### Multiresistance among ESC-resistant *Shigella* isolates

Among the 356 *Shigella* isolates, 69 (36 ESC-resistant *S. flexneri*, 23 cefotaxime susceptible *S. flexneri*, 6 ESC-resistant *S. sonnei* and 4 cefotaxime susceptible *S. sonnei*) were multidrug resistant (MDR, resistant to 3 or more classes of antimicrobial agents), and the multi-resistance was more frequent among *S. flexneri* isolates than among *S. sonnei* isolates (Table 2).

### Production of ESBL and AmpC

The phenotypic confirmatory test displayed that 96.0% (190/198) ESC-resistant isolates were ESBLs positive (Table 3). Of 8 cefoxitin resistant or intermediate isolates, 2 were the three-dimensional extract test positive.

**Table 3:** **Distribution of genotypes of ESBL and AmpC in 198 ESC-resistant *Shigella *** **isolates**

**ESBL phenotypic confirmatory test (n)**	**Genotype**		***S. flexneri *** **(n = 58)**	***S. sonnei *** **(n = 140)**	**Total (n = 198)**
	**ESBL or AmpC group**	**ESBL or AmpC gene**			
Positive (n = 190)	CTX-M-1	CTX-M-15	5	11	16
		CTX-M-28	2	6	8
		CTX-M-57	7	2	9
		CTX-M-22	3	2	5
	CTX-M-9	CTX-M-14	16	69	85
	TEM	TEM-1	2	4	6
	OXA	OXA-30	4	11	15
	CTX-M-1+CTX-M-9+OXA	CTX-M-15+CTX-M-14+OXA-30	1	0	1
	CTX-M-1+ OXA+TEM	CTX-M-22+OXA-30+TEM-1	0	2	2
		CTX-M-57+ OXA-30+TEM-1	9	0	9
	CTX-M-1+ TEM	CTX-M-57+ TEM-1	2	0	2
	CTX-M-1+ OXA	CTX-M-57+ OXA-30	1	1	2
		CTX-M-15+ OXA-30	2	8	10
	CTX-M-9+ OXA+TEM	CTX-M-14+ OXA-30+ TEM-1	1	3	4
	CTX-M-9+ OXA	CTX-M-14+ OXA-30	2	6	8
	CTX-M-9+ TEM	CTX-M-14+ TEM-1	0	6	6
	Not detect the ESBL or AmpC genotype		0	2	2
Negative (n = 8)	CTX-M-9+CIT	CTX-M-14+CMY-2	0	1	1
	CTX-M-1+DHA+OXA	CTX-M-15+OXA-30+DHA-1	1	0	1
	Not detect the ESBL or AmpC genotype		0	6	6

### Distribution of ESBL and AmpC genes

Of the 198 ESC-resistant isolates, 105 (53.0%) were positive for *bla*_*CTX-M-14*_, 28 (14.1%) for *bla*_*CTX-M-15*_, 22 (13.6%) for *bla*_*CTX-M-57*_, 8 (4.0%) for *bla*_*CTX-M-28*_, 7 (3.3%) for *bla*_*CTX-M-22*_, 52 (26.3%) for *bla*_*OXA-30*_, 1 (0.5%) for *bla*_*CMY-2*_, and 1 (0.5%) for *bla*_*DHA-1*_; 28 (14.1%) isolates carried non-ESBL gene *bla*_*TEM-1*_; 8 (4.0%) strains were negative for all β-lactamase genes detected. No isolate hosted *bla*_*SHV*_, *bla*_*ACC*_, *bla*_*EBC*_ and *bla*_*FOX*_ genes. And 44 (23.2%) isolates were found to harbor ≥2 β-lactamase genes (Table 3).

### Distribution of virulence genes

All ESC-resistant *Shigella* isolates harbored the *ial*, *ipaH* and *virA* genes. The gene *sen* existed in 99.0% *Shigella* isolates. The genes *set1A* and *set1B* were more prevalent in *S. flexneri* strains than in *S. sonnei* isolates (*p* < 0.05) (Table 4). Of 198 ESC-resistant *Shigella* strains, 123 (*S. sonnei* isolates accounting for 92.7%, 114/123) possessed the most prevalent virulence gene combination of *ia1* + *ipaH* + *virA* + *sen*, followed by the combination of *ia1* + *ipaH* + *virA* + *set1A* + *set1B* + *sen* (*S. flexneri* isolates accounting for 86.8%, 46/53) (Table 4).

**Table 4:** **Prevalence and combination of virulence genes among 198 ESC-resistant *Shigella *** **isolates**

**Isolate**	**Serotype**	**No. (%) of isolates that produced the following:**						**No. (%) of isolates that contained the following combination of genes**				
		***ial***	***ipaH***	***virA***	***set1A***	***set1B***	***sen***	**I**	**II**	**III**	**IV**	**V**
*S. flexneri* (n = 58)	1a (n = 2)	2 (3.4)	2 (3.4)	2 (3.4)	0 (0.0)	0 (0.0)	2 (3.4)	0 (0.0)	2 (3.4)	0 (0.0)	0 (0.0)	0 (0.0)
	2a (n = 37)	37(63.8)	37 (63.8)	37 (63.8)	36 (62.1)	36 (62.1)	37 (63.8)	0 (0.0)	1 (1.7)	0 (0.0)	0 (0.0)	36 (62.1)
	2b (n = 2)	2 (3.4)	2 (3.4)	2 (3.4)	0 (0.0)	0 (0.0)	2 (3.4)	0 (0.0)	2(3.4)	0 (0.0)	0 (0.0)	0 (0.0)
	2c (n = 5)	5 (8.6)	5 (8.6)	5 (8.6)	0 (0.0)	0 (0.0)	5 (8.6)	0 (0.0)	5 (8.6)	0 (0.0)	0 (0.0)	0 (0.0)
	4c (n = 12)	12(20.7)	12 (20.7)	12 (20.7)	10 (17.2)	10 (17.2)	12 (20.7)	0 (0.0)	2 (3.4)	0 (0.0)	0 (0.0)	10 (17.2)
		58 (100)	58 (100)	58 (100)	46 (79.3)	46 (79.3)	58 (100)	0 (0.0)	12 (20.7)	0 (0.0)	0 (0.0)	46 (79.3)
*S. sonnei* (n = 140)		140(100)	140(100)	140(100)	12 (8.6)	19 (13.6)	138(98.6)	2 (1.4)	114 (81.4)	5 (3.6)	12 (8.6)	7 (5.0)
*S. flexneri* vs *S. sonnei* (*p* value)		NA	NA	NA	<0.001	0.003	0.360	0.364	<0.001	0.603	0.021	<0.001
Total		198(100)	198(100)	198(100)	58 (29.3)	65 (32.8)	196 (99.0)	2 (1.0)	123(62.1)	8 (4.0)	12 (6.1)	53 (26.8)

*p* < 0.05 were considered statistically significant.

I, *ia1* + *ipaH* + *virA*; II, *ia1* + *ipaH* + *vir* + *sen*; III, *ia1* + *ipaH* + *virA* + *setlA* + *sen*; IV, *ia1* + *ipaH* + *virA* + *setlB* + *sen*; V, *ia1* + *ipaH* + *virA* + *setlA* + *setlB* + *sen.*

NA, not available.

### ERIC-PCR

The 58 ESC-resistant *S. flexneri* isolates were classified into 8 types by ERIC-PCR. The distribution of isolates based on this epidemiological analysis was as follows: 26 isolates in type A, 11 isolates in type B, 5 isolates in type C, 4 isolates each in type D and type E, 3 isolates each in type F and type G, and 2 isolates in type H. Among the 140 ESC-resistant *S. sonnei* isolates, 13 different genotypes (type A to M) were obtained. The major genotype was type A (66 isolates), followed by type B (19 isolates), type C (12 isolates), type D (8 isolates), type E (7 isolates), type F (7 isolates), type G (6 isolates), type H (6 isolates), type I (3 isolates), type J (2 isolates), type K (2 isolates), type L (1 isolates) and type M (1 isolates).

## Discussion

This investigation reported the prevalence and resistance of ESC-resistant *Shigella*, and the molecular analysis of cephalosporin resistance genes and virulence determinants in clinical isolates from Xiaoshan District, Hangzhou, China collected over a period of 5 years. In the economic undeveloped regions, *S. flexneri* is the most frequently isolated *Shigella* species. A similar situation also exists in China, according to previous data [2,4,6,16-18]. However, in our study *S. sonnei* was the most common cause of bacterial dysentery, which was consistent with the findings in industrialized countries. In recent years, the data from Kaengkhoi District of Thialand, Ho Chi Minh City of Vietnam, South Korea, Taiwan and the East, North and Northeast regions of China, which are the newly industrialized regions, also displayed a striking species shift from *S. flexneri* to *S. sonnei*[18-22]. Therefore, the species transition of shigellosis in the present study may be related to the economic growth in Xiaoshan District, suburban of Hangzhou city with higher economic indicators. Of course, other factors may also play a role and need further researches.

Through the resistance data analysis, we found that more than half of the *S. flexneri* and *S. sonnei* isolates were resistant to ESC (cefotaxime). Data from 8 Asian countries showed a high prevalence of resistance to the frontline antibiotics AMP (53.0%) and SXT (81.0%) among *Shigella* isolates [21]. However, the resistance rates to the both drugs among all our *Shigella* isolates were more higher (AMP, 97.8%; SXT, 85.4%), and were consistent with the data from other investigations, mainland China [6,16,17]. The data from Table 2 showed that SAM was not appropriate for the treatment of diarrhea caused by *S. flexneri*, regardless of the strains with ESC-resistance (96.6%) or susceptibility (85.2%); On the contrary, it can be used to prescribe for *S. sonnei* infections, especially for the cefotaxime susceptible strains. Although the resistance rate of *Shigella* to PIP was high (83.7%) in this study, TZP was of very high anti-*Shigella* activity (Table 2).

If the inhibition zone diameter of CAZ is ≥21 mm or that of FEP ≥18 mm, the two antibiotics can be reported susceptibility for enterobacteriaceae, irrespective of the isolates producing ESBLs or not, according to the CLSI [10]. In all *Shigella* isolates studied, a similar results of resistance to CAZ and FEP (16.6% and 18.5%) were observed, which were higher than that reported by Yang et al. (5.2% and 6.5%) [16]. However, the resistance rates in Table 2 indicated that the two antibiotics were more suitable for the empiric therapy of the infection of ESC-resistant *S. sonnei* than that of ESC-resistant *S. flexneri* infection.

Fluoroquinolones are the popular antibiotics for the treatment of serious shigellosis in both adults and children. Research results from Gu et al. [23] showed the resistance rate to CIP was 29.1% between 2007 and 2009 in the Asia-Africa area. Data from Henan Province, China displayed that 21% and 79% of *S. flexneri* strains showed high- or low-level resistance to CIP, respectively [6]. Yang et al. [16] reported that 27.9% and 9.7% *Shigella* were resistance to CIP and LEV, respectively, in Anhui province, China. In our study, a similar resistance rate to CIP (24.2%) and a higher resistance rate to LEV (16.0%) were exhibited (Table 2). Among the fluoroquinolone resistant isolates, 95.3% (82/86, resistance to CIP) and 89.5% (51/57, resistance to LEV) strains belonged to *S. flexneri*. The possible cause was that *S. flexneri* isolates often possessed plasmid-mediated quinolone resistance (PMQR) determinants or mutations in quinolone resistance-determining regions (QRDR) of gyrase and topoisomerase genes. This situation had been described in isolates from other regions of China by Zhang et al. [4], Zhu et al. [24] and Pu et al. [25].

So far, at least 109 variants of CTX-M enzymes (CTX-M-1 to 124) have been described. Of these CTX-Ms, 19 variants (CTX-M-15, 16, 19, 23, 25, 27, 32, 35, 37, 40, 42, 53, 54, 55, 57, 58, 62, 64, 82, 93) exhibit the increased hydrolysis activity against ceftazidime, and the others display a much higher rate of hydrolysis of cefotaxime than ceftazidime [26]. CTX-M-15 is the most usually detected CTX-M variant that hydrolyze ceftazidime at high level in enterobacteriaceae [26]. In this study, 28 *bla*_*CTX-M-15*_ positive ESC-resistant *Shigella* isolates were all resistant to ceftazidime (data not shown). No other CTX-M variant genes mediating high level ceftazidime resistance were found (Table 3). In the *bla*_*CTX-M*_ genes with higher catalytic efficiencies against cefotaxime than ceftazidime, *bla*_*CTX-M-14*_ was the most prevalent one (53.0%), and coincides with the data published worldwide in clinically important pathogens [26]. OXA-30 belongs to the class D oxacillinase group III, and mediates resistance to cefepime but not ceftazidime [27]. Unfortunately, 52 (26.3%) of our ESC-resistant *Shigella* isolates hosted *bla*_*OXA-30*_, and 12 of them carried *bla*_*CTX-M-15*_ concomitantly and conferred resistance to cefotaxime, ceftazidime and cefepime (Table 3). In the past decade, an emergence of ESBL-producing *Shigella* spp. carrying different types of ESBL genes has been described in different countries and regions [28]. However, only a few studies have reported in the world the existence of AmpC β-lactamases encoded by *bla*_*CMY-2*_ or *bla*_*DHA-1*_ in *Shigella* spp. [4,25,28-31]. In this study, we also found 2 AmpC β-lactamase producers with *bla*_*DHA-1*_ and *bla*_*CMY-2*_ in the three-dimensional extract test positive *Shigella* strains. The *bla*_*CMY-2*_ and the *bla*_*DHA-1*_ existed in 1 *S. flexneri* with *bla*_*CTX-M-14*_ and 1 *S. sonnei* with *bla*_*CTX-M-15*_ and *bla*_*OXA-30*_, respectively (Table 3).

In this study, we detected several pathogenic genes (*ial*, *ipaH*, *set1*, *sen* and *virA*) for 198 ESC- resistant *Shigella* isolates (Table 4). It has been shown that *ial* takes responsibility for penetration of epithelial cell by *Shigella* and *ipaH* also for spread from cell to cell [32,33]. All *Shigella* species studied were positive for the *ipaH* as expected because this gene exists in multiple copies on both the chromosome and the plasmid of *Shigella*. Conversely, the *ial* gene is exclusively located on the plasmid and was only detected in some *Shigella* isolates [8]. Indeed, less frequent examination of *ial* gene had been described by Luscher and Altwegg [34], Kingombe et al. [35] and Thong et al. [36]. However, this gene was found in all our ESC-resistant *Shigella* strains. Another virulence factor VirA is involved in the uptake, motility, and cell to cell transmission of *Shigella* within the human host. It is an essential virulence factor in *Shigella* disease pathogenesis [9]. The positive rate of *virA* implied all the isolates of our collection might have the ability (Table 4). The *set1* chromosomal gene encodes *Shigella* enterotoxin 1 (ShET-1, composed of one A and five B subunits), which is generated by *S. flexneri* (mainly in type 2a) and not found in other *Shigella* spp. [8,12,36-41]. The *sen* gene encoding *Shigella* enterotoxin 2 (ShET-2) is carried on a 140 MDa virulence plasmid. And the *sen* is present in all *Shigella* species. The both toxins are deemed to play a part in the clinical manifestation of shigellosis [8]. In our study, 79.3% of ESC-resistant *S. flexneri* strains were found to be *set1A* and *set1B* positive (62.1% isolates were serotype f2a, Table 4), and this is agreement with the previous results; however, 17.1% (24/140) ESC-resistant *S. sonnei* isolates also carried *set1A* and/or *set1B* genes (Table 4). The *set1A* and *set1B* genes are located on the *she* pathogenicity island (PAI), a chromosomal, laterally acquired, integrative element of *S. flexneri*[37]. Integrase-mediated excision can occur for the *she* PAI, and result in the formation of a circular excision product, which is a substrate for lateral transfer processes e.g. conjugation, packaging into phage particles and recombinase-mediated integration into the chromosome [42]. This may be the cause that the two determinants can be found in our ESC-resistant *S. sonnei* isolates studied. And the deficiency of *set1A* or *set1B*, or the existence of point mutations in the primer binding sites may be the possible explanation for the both genes not coexisting in some ESC-resistant *S. sonnei* isolates (Table 4). Furthermore, we found that the *S. flexneri* isolates with *set1* were more resistance to CIP, LEV (p < 0.001, each) and FEP (p = 0.019) than those without *set1*; for *S. sonnei*, the *set1* positive isolates were more likely resistance to SAM (p < 0.001), CIP, LEV (p < 0 .001, each) and FEP (p = 0.002), and more likely sensitivity to CAZ (p = 0.005) than *set1* negative ones (data not shown). Nevertheless, we do not think there was correlation between *set1* and the antibiotics resistance, because none of reports have described currently that the invasion genetic elements carrying virulence genes known contain resistance determinants simultaneously in *Shigella* spp.. These resistance differences may be only due to the spread of resistant plasmids among the different strains.

The results of ERIC-PCR typing showed that most of the cases of ESC-resistant *S. flexneri* and *S. sonnei* infections were caused by several identical strains, respectively [for *S. flexneri* isolates, the percentage of type A and type B accounting for 63.8% (37/58); for *S. sonnei*, type A, type B and type C encompassing 69.3% (97/140) of the isolates]. This indicates that clonal dissemination was likely to contribute most to the spread of ESC-resistant *S. flexneri* and *S. sonnei* within the region studied. Of 46 ESC-resistant *S. flexneri* isolates with the second largest number of the virulence gene composition (*ia1* + *ipaH* + *virA* + *setlA* + *setlB* + *sen*) being resistant to 4 to 8 antibiotics, 60.3% belonged to type A (43.1%) and type B (17.2%). Of 114 ESC-resistant *S. sonnei* isolates with the first largest number of the virulence gene composition (*ia1* + *ipaH* + *vir* + *sen*) being resistant to 3 to 6 antibiotics, 71.1% belonged to type A (57.9%) and type B (13.2%), but isolates that hosted the *set1* gene were more heterogeneous in ERIC-PCR pattern.

In the present study all the *Shigella* strains were isolated from intestinal clinic patients. No patients had been hospitalized or died following episodes of shigellosis. According to the clinical reports, early stage of shigellosis the patients infected by ESBL genes positive isolates were not more severe than those of the patients infected by ESBL genes negative isolates. However, most of these patients had longer course of treatment, because physicians used to treat diarrhea by prescribing cefotaxime or ceftriaxone (particularly for children) in the region studied. When treatment failed, other drugs (such as fluoroquinolone or β-lactamase inhibitors) would be used as a substitute for continuing treatment. In addition, all the patients with *Shigella* infection in the study were treated with antibiotics, therefore, we had no relevant data to compare the course of disease treated using antibiotics with that of disease treated without antibiotics.

## Conclusions

This investigation found a striking difference from other regions of China with relatively low economic status in the distribution of species, from *S. flexneri* to *S. sonnei*, and a high prevalence of ESC-resistance among *Shigella* isolates. Antibiotic susceptibility testing displayed ESC-resistant *S. flexneri* isolates were much more resistant than ESC-resistant *S. sonnei* isolates. The analysis of extended-spectrum cephalosporin resistance genes showed the spread of the *Shigella* harboring a combination of genes *bla*_*CTX-M-15*_ and *bla*_*OXA-30*_. This combination may be a serious threat to infection treatment because it is spread by conjugation and it displays high levels of resistance to cefotaxime, ceftazidime and cefepime. We also described the prevalence of virulence genes in ESC-resistant *Shigella*, which may provide clues about the pathogenicity difference of this type of bacteria. ERIC-PCR typing supported the existence of specific clones responsible for the ESC-resistant *S. flexneri* and *S. sonnei* infection cases in the region studied. In China, especially in some undeveloped regions, there were limited and sporadic data on the epidemiology of *Shigella* spp. Therefore, we suggest the Ministry of Health National Antimicrobial Resistance Investigation Net (Mohnarin) to reinforce the surveillance for the development of antibiotic resistance and take measures to control shigellosis in China.

## Abbreviations

ESC: Extended-spectrum cephalosporin; ESBL: Extended-spectrum beta-lactamase; S. flexneri: *Shigella flexneri*; S. sonnei: *Shigella sonnei*; CLSI: Clinical laboratory standards institute; PCR: Polymerase chain reaction; ERIC-PCR: Enterobacterial repetitive intergenic consensus sequence polymerase chain reaction; SPSS: Statistical package for social sciences.

## Competing interests

The authors declare no conflict of interests.

## Authors’ contributions

CLZ performed the experiments; QZL designed the study, analyzed the clinical data and wrote this manuscript; JW, XC, LMS and YYG collected the clinical samples. All authors read and approved the final manuscript.

## Pre-publication history

The pre-publication history for this paper can be accessed here:

http://www.biomedcentral.com/1471-2334/14/260/prepub
